# Sundown syndrome and symptoms of anxiety and depression in hospitalized elderly

**DOI:** 10.1590/1980-57642016dn11-020008

**Published:** 2017

**Authors:** Marcello Weynes Barros Silva, Rilva Lopes Sousa-Muñoz, Heitor Cabral Frade, Priscilla Alencar Fernandes, Andrêssa de Oliveira Magalhães

**Affiliations:** 1 Graduado em Medicina pela Universidade Federal da Paraíba e Aluno Bolsista da Pesquisa; 2 Doutora pela Universidade Federal da Paraíba e Orientadora da Pesquisa. Departamento de Medicina Interna; 3 Graduando em Medicina pela Universidade Federal da Paraíba; 4 Fernandes, Priscila; Graduando em Medicina pela Universidade Federal da Paraíba. Graduando em Nutrição pelo Instituto Superior de Teologia Aplicada; 5 Graduando em Nutrição pelo Instituto Superior de Teologia Aplicada.

**Keywords:** dementia, biological rhythms, cognitive neuroscience, demência, ritmos biológicos, neurociência cognitiva

## Abstract

Sundown syndrome is characterized by the sudden appearance of neuropsychiatric symptoms such as agitation, confusion and anxiety in a chronologic fashion, usually during late afternoon or early evening.

**Objective:**

To evaluate the prevalence of sundown syndrome in university hospital wards and its relationship with anxiety/depression symptoms, cognitive decline, and clinical and demographic variables.

**Methods:**

We evaluated 70 patients admitted to the Lauro Wanderley University Hospital (HULW), João Pessoa-PB, Brazil. Data collection instruments were the Confusion Assessment Method (CAM), the Mini-Mental State Exam (MMSE) and the Hospital Anxiety and Depression Scale (HADS).

**Results:**

Mean patient age was 68.4±6.4 years, 55.7% were male, 67.1% were illiterate or had incomplete primary education. It was observed that 14.3% of patients had delirium, 15.7% had cognitive deficits, while 21.4% and 18.6% had anxious and depressive symptoms, respectively. The age of patients with delirium (71.9±8.7) was significantly higher than those without (67.8±5.8). At 95% confidence, there was a significant difference in the groups with and without delirium for the MMSE and HADS-D scales.

**Conclusion:**

We verified the occurrence of delirium compatible with the sundown syndrome and associated with depressive symptoms and cognitive deficit, with no apparent relationship with infectious processes or fever, number of drugs used, hospital stay or anxious symptomatology.

## Abstract (pt)

A síndrome de Sundown é caracterizada pelo aparecimento súbito de sintomas neuropsiquiátricos como agitação, confusão e ansiedade de forma cronológica, geralmente no final da tarde ou no início da noite.

**Objetivo:**

Avaliar a prevalência da síndrome do por do sol em enfermarias de um hospital universitário e sua relação com sintomatologia depressivo-ansiosa, déficit cognitivo e variáveis clínicas e demográficas.

**Métodos:**

Foram avaliados 70 pacientes admitidos nas enfermarias Hospital Universitário Lauro Wanderley (HULW), João Pessoa-PB, Brasil. Os instrumentos de coleta de dados foram a Escala de Avaliação de Quadros Confusionais (*Confusion Assessment Method* - CAM), o Mini-Exame do Estado Mental (MEEM) e a Escala Hospitalar de Ansiedade e Depressão (HADS).

**Resultados:**

A média de idade de 68,4±6,4 anos, 55,7% do sexo masculino, 67,1% não alfabetizados ou com instrução fundamental incompleta. Observou-se que 14,3% dos pacientes tinham *delirium*, 15,7% tinham déficit cognitivo, enquanto 21,4% e 18,6% apresentavam sintomas ansiosos e depressivos, respectivamente. A idade dos pacientes com *delirium* (71,9±8,7) foi significativamente maior que a dos que não apresentavam este quadro (67,8±5,8). Com 95% de confiança, há diferença significativa nos grupos com e sem delirium em relação das escalas do MEEM e do HADS-D.

**Conclusão:**

Verificamos a ocorrência de *delirium* compatível com a síndrome do pôr do sol e associado com sintomas depressivos e déficit cognitivo, sem relação aparente com processo infeccioso ou febre, número de medicamentos em uso, permanência hospitalar e sintomatologia ansiosa.

## INTRODUCTION

Sundown syndrome is characterized by the sudden appearance of neuropsychiatric symptoms such as agitation, confusion and anxiety in a chronologic fashion, usually during late afternoon or early evening, between 4pm and 6pm. It commonly affects institutionalized or cognitively impaired individuals, but may also affect elderly inpatients.^1^ There is still no consensus on its operational definition, due to the lack of clinical evidence in the literature. It remains a descriptive term rather than a psychiatric diagnosis which occurs mainly in patients with decreased cognition or institutionalized elderly, but can also occur in hospitalized elderly in general wards.^2^ There is evidence that the syndrome affects 2.4% to 25% of patients with dementia, but it seems to affect virtually all patients with some degree of cognitive impairment and also some cognitively normal patients.^1^

One of the main problems affecting hospitalized elderly patients is an acute change in consciousness and orientation, better known as acute mental confusion or *delirium*, which takes place especially when there is lack of sensory stimulation. This condition is a complication seen in at least 25% to 60% of hospitalized elderly.^3,4^ *Delirium* is a mental disorder characterized by acute onset, fluctuating course and changes in consciousness, memory, thought, perception and behavior. It may present as hyperactive, hypoactive or mixed in up to 50% of elderly inpatients, many of whom have preexisting dementia.^5^

Sundowning syndrome refers to a state of acute mental confusion and behavioral change that takes place at the end of the day and into the night.^6,7^ It is equivalent to a *delirium* that is precipitated by diminished illumination, and can also be confused with depression or dementia. The difference lies in the fact that its disruptive behavior characteristically presents at sunset or evening. As natural light diminishes and increased shadows appear, symptoms can further aggravate. In addition, it has been shown that the hospital care team may not perceive changes in illumination between different parts of the hospital. Other clinical features are mood changes, distrust and visual and auditory hallucinations.^1^

Other precipitating factors have been described, including polypharmacy changes in the environment, which may have a role in circadian rhythm. Few studies have addressed the sundown syndrome, whose prevalence varies between 10% and 20% within institutionalized elderly.^1,2^

The diagnosis of sunset syndrome is purely clinical and involves a wide range of cognition, mood and behavior abnormalities, with temporal pattern of expression, in the late afternoon or evening.^1^ It is not a formally recognized psychiatric diagnosis, but rather a syndrome diagnosis.

Besides illumination, social isolation and clinical worsening have been linked to some sundowning-related symptoms, such as anxiety and depression.^2^ These symptoms may further aggravate preexisting organic and cognitive conditions. Furthermore, mood symptoms are commonly present within hospitalized individuals. A study conducted at Lauro Wanderley University Hospital (HULW), showed that 23% of elderly patients admitted to medical wards had clinically significant depressive symptoms.^8^

Another psychological disorder that affects the elderly associated with depressive symptoms and cognitive impairment is anxiety, characterized by a transient emotional state that involves unpleasant feelings of tension, anguish and suffering.^9^

Illness has a particular meaning for the elderly, since it brings with it the fear of physical dependence, feelings of hopelessness and perception of finitude, which are more prominent when they are hospitalized. Inpatients of all ages tend to feel intimidated, which ultimately contributes to adverse emotional reactions such as depressive and anxiety symptoms.^10^ These are common triggering factors of functional decompensation at psychological and cognitive levels.

The sundowning phenomenon is also closely related to circadian rhythm abnormalities. These disturbances are more prominent and disabling in patients with dementia and delirium, when compared with healthy elderly. Deterioration of circadian rhythm in these patients is probably multifactorial, caused by the neurodegenerative process, pathological changes in the retina and hypothalamic suprachiasmatic nucleus, and environmental factors.^11^

The primary objective of this study was to verify the prevalence of delirium at the end of the afternoon in the elderly hospitalized in the wards of a university hospital. The secondary objectives were to evaluate the relationship between delirium and symptoms of anxiety and depression, besides other clinical and demographic variables. The relevance of the theme stems from the high morbidity of the sunset syndrome and its prevalence not being known in the elderly hospitalized in secondary care centers in Brazil.

## METHODS

**Study model, site and period of observation.** We performed a cross-sectional study using a quantitative approach, from September 2014 to August 2015. Data was collected in surgical, internal medicine and infection wards at the HULW, at the Federal University of Paraíba (UFPB). The patients recruited had at least 24 hours of hospitalization in the wards, and the application of questionnaire at bedside was performed between 17:30 and 19:00.

The HULW is a tertiary referral hospital in João Pessoa/PB and for nearby cities of Paraiba, Pernambuco and Rio Grande do Norte. The hospital receives patients from several regional health services in its 212 beds, at least 70 in the internal medicine ward (IMW) and 20 in the surgical ward (SW).

In the IMW, 800 patients are admitted annually, predominantly middle-aged and elderly patients, carriers of various diseases and requiring curative or hospital rehabilitation. Elderly inpatients in the service characteristically have low income and education, comprising a large portion of the admitted patients, 36.5% of total admissions on 2011.^9^

**Sample and strategy of action.** We used a convenient method to recruit 70 patients older than 60 years, admitted to HULW over the study period. According to the Brazilian Elderly Statute (Law no. 10.741, of October 1st, 2003), individuals over 60 years old are considered elderly.^12^

The following inclusion criteria were adopted: a) age 60 or older; b) both sexes; (c) admitted to HULW for medical or surgical treatment in the wards; (d) able to give their informed consent to participate in the study. Exclusion criteria were: (a) liver disease or decompensated kidney disease; (b) patients with severe medical condition; (c) presence of any communication disturbance, such as aphasia, coma, speech difficulty or severe hearing impairment.

On a standardized form, the following demographic and clinical variables were assessed: (A) demographic variables: age, sex, education; (B) clinical variables definitive diagnosis which led to hospitalization; number and type of medications during hospitalization; number of hospitalizations during the last year; cognitive impairment history (patient referral, family and/or both, sufficient deficit to affect the patient’s ability to recognize people, carry out activities of daily living or walk without aids); record of axilla temperature at the time of the interview.

Complaints of hearing loss and visual impairment were assessed by self-report of patients and their caregivers, and ultimately through direct questions about hearing and vision loss. Questions that allow identification of elderly with hearing loss involved speech comprehension in quiet and noisy environments, which have demonstrated high sensitivity values.^13^ Diagnoses were grouped according to the International Statistical Classification of Diseases and Related Health Problems, 10th revision (ICD-10).^14^ Data collection was performed by three previously trained graduate students in medicine at the UFPB.

The main data collection instruments were three standard scales for assessing depressive symptoms, anxiety and cognitive:

*(A) CAM Algorithm.* Confusion Assessment Method - CAM: an instrument created for the diagnostic evaluation of delirium,^15^ validated in Brazil.^16^ In the validation study, CAM was applied to 100 hospitalized elderly to objectively assess delirium diagnosed by DSM -IV (Diagnostic and Statistical Manual of Mental Disorders, 4th edition). The authors found high sensitivity (94.1%) and specificity (96.3%).^15^ The inter-observer concordance of this scale was also high (Kappa statistic 0.91) and internal consistency was adequate (Cronbach’s alpha=0.84).^17^

Diagnosis of delirium by CAM is based on the first four factors (clinical features) and requires the presence of acute onset of symptoms and disorders of thought or changes in level of consciousness. For the positive screening of delirium by CAM, factors 1 and 2 must be present (acute onset of symptoms and attention disorder) and the patient must also exhibit symptoms of delirium, with the presence of factors 3 and 4 (disorganized thinking and altered level of consciousness).

*(B) Mini-Mental State Examination (MMSE).* This instrument provides information on different cognitive parameters, with questions grouped into seven categories, each designed to evaluate specific cognitive functions. It evaluates temporal orientation, spatial orientation, three word registration, attention and calculation, three word recall, language and visual construction capacity.^18^ The MMSE score can range from a minimum of zero, which indicates the lowest degree of cognitive performance of individuals, up to a maximum of 30 points. The following cutoffs were adopted: 13 for illiterates, 18 for those with primary or secondary education and 26 for those with at least tertiary education.^19^

*(C) Hospital Anxiety and Depression Scale (HADS).* Prepared by Zigmond and Snaith (1983),^20^ the scale consists of 14 items, seven related to anxiety. It has also been translated and validated for use in Brazil.^21^ Each of the items can be scored from 0 to 3, with a maximum score of 21 points. the cutoff points adopted for both subscales are: HADS-A - anxiety - without anxiety 0-8, with anxiety ≥ 9; HADS-D - depression - without depression 0-8, with depression ≥ 9. Scores > 8 on the HADS-D subscale were considered suggestive of depression and classified as clinically significant depressive symptoms, with sensitivity and specificity of 80% and predictive value of 70%.^22^ Cronbach’s alpha for the HADS-A was reported in the literature as ranging from 0.68 to 0.93 (mean 0.83) and for the HADS-D from 0.67 to 0.90 (mean 0.82).^23^

**Statistical analysis.** For descriptive statistics, absolute and relative frequencies of qualitative variables were assessed, as well as mean and standard deviation of quantitative variables.

For inferential statistics, dichotomous variables were analyzed by the Chi-square test while ordinal and interval variables were analyzed by Kruskal-Wallis and Mann-Whitney tests, respectively. Spearman correlation was used to assess correlations between ordinal and interval variables. The level of statistical significance was 5%. The statistical program SPSS 20.0 for Windows was employed in the statistical analysis.

**Ethical aspects.** The project was approved by the Research Ethics Committee of the HULW-UFPB. All patients who agreed to participate signed the Informed Consent Agreement approved by the CEP under CAAE n° 31039914.9.0000.5183. Researchers assured respondents that both subjects and their responses would be kept anonymous throughout the whole study.

## RESULTS

Between September 2014 and July 2015, 70 patients consecutively admitted to the medical, surgical and infectious diseases wards of the HULW were selected for the study. Age of the 70 patients ranged from 60 to 86 years, with a mean of 68.4 (±6.4) years without significant difference between genders (55.7% male) and 67.1% were illiterate or had not completed primary level education (Table 1).

**Table 1:** Demographic characteristics of elderly patients hospitalized in the University Hospital Lauro Wanderley between September 2014 and July 2015 (n=70).

Variables		Frequency	
		**F**	**%**
Genre	Male	39	55.7
	Female	31	44.3
Age	60-69 years	43	61.4
	70-79 years	24	34.3
	80 or over	3	4.3
Educational level	Illiterate	21	30.0
	Incomplete primary	26	37.1
	Complete primary	5	7.1
	Complete secondary	7	10.0
	Higher	3	7.1
	Not informed	6	8.6

Digestive, circulatory and respiratory diseases were together (38.7%) primarily responsible for admissions of patients in this study sample (Table 2). The duration of hospitalization until the time of interview ranged from 1 to 38 days with a mean of 9.5 (±8.8) days. The mean number of drugs used on the assessment day was 7.15 (±3.2), and the number of hospitalizations in the last year ranged from one to six, with a mean of 0.6 (±1.2).

**Table 2:** Distribution of elderly patients in wards of Lauro Wanderley University Hospital between September 2014 and July 2015 according to diagnosis that led to hospitalization, as defined by categories of ICD-10 (n=70).

Diagnosis categorized by ICD-10*	Frequency	
	**f**	**%**
Diseases of the digestive system	9	12.9
Diseases of the circulatory system	9	12.9
Respiratory diseases	9	12.9
Infectious and parasitic diseases	8	11.4
Diseases of the genitourinary apparatus	7	10.0
Endocrine disease	4	5.7
Abnormal findings not elsewhere classified	4	5.7
Others	4	5.7

* ICD-10: International Statistical Classification of Diseases and Related Health Problems, 10th revision.

Clinical variables such as sensory deficit (auditory and visual), the presence of diagnosed infection, fever and mechanical containment in bed are shown in Table 3.

**Table 3:** Clinical variables of elderly patients in wards of Lauro Wanderley University Hospital between September 2014 and July 2015 (n=70).

Variables	Frequency	
	**f**	**%**
Presence of infection	26	37.7
Presence of fever	3	4.3
Restraint in bed	6	8.6
Hearing loss	10	14.3
Low visual acuity	32	45.7

Age and MMSE scores were higher in patients hospitalized with delirium than in those without mental confusion, but this difference was statistically significant only in relation to MMSE scores (p=0.001).

The mean scores on the MMSE differed according to the educational level of the patients, with progressively lower scores as the level of education decreased. Duration of hospital stay did not differ significantly for presence of delirium (p=0.178).

**Figure 1:**
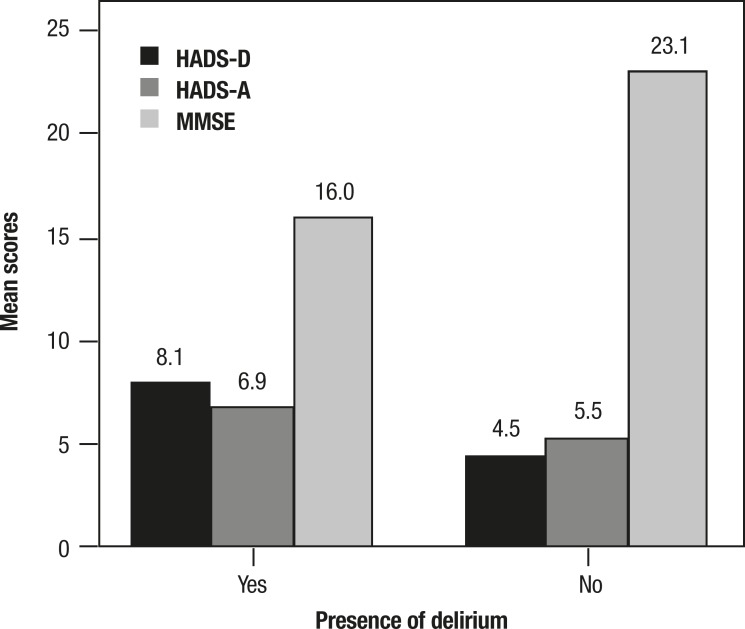
Comparison of scores on assessment scales for elderly patients with and without delirium (n=70) hospitalized at the University Hospital Lauro Wanderley.

Scores obtained on the HADS-D and MMSE varied significantly among patients with delirium and those without the condition, as defined by the CAM. The scores on the HADS-A subscale did not differ significantly between older adults with and without delirium (Figure 1). Co-occurrence of significant depressive symptoms and delirium was observed in 5.7% of patients, while co-occurrence of significant anxiety symptoms and delirium was 4.3%.

There was no gender difference for the occurrence of delirium (p=0.961). There was no statistically significant difference between men and women in relation to scores on the HADS-A (p=0.094) and HADS-D (p=0.874).

The scores on the subscales of the HADS were significantly higher in patients with mechanical restraint to bed for both the HADS-D (p=0.02) and HADS-A (p<0.001). The presence of delirium did not differ in relation to whether or not the patient was mechanically contained.

There was no difference in depressive and anxiety scores in relation to hearing loss, low visual acuity, duration of hospital stay, number of medications, number of hospitalizations in the previous year or age.

**Figure 2:**
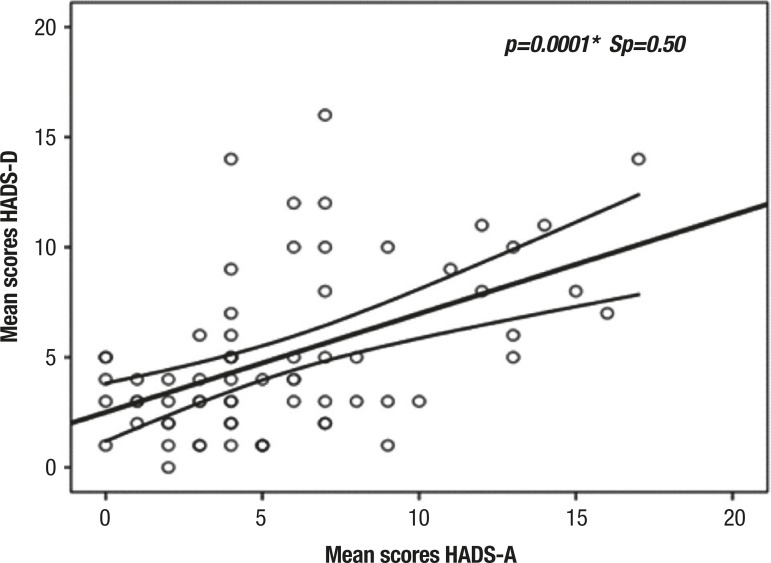
Correlation between mean scores on subscale of depressive symptoms (HADS-D) and subscale of anxiety symptoms (HADS-A) in elderly patients hospitalized at the University Hospital Lauro Wanderley (n=70).

There was a statistically significant moderate linear positive correlation (p<0.05, Sp= +0.5) between the subscale scores of depressive symptoms and the subscale scores of anxiety symptoms on the HADS, indicating that the higher the score on the HADS-D, the higher the score achieved on HADS-A (Figure 2).

**Figure 3:**
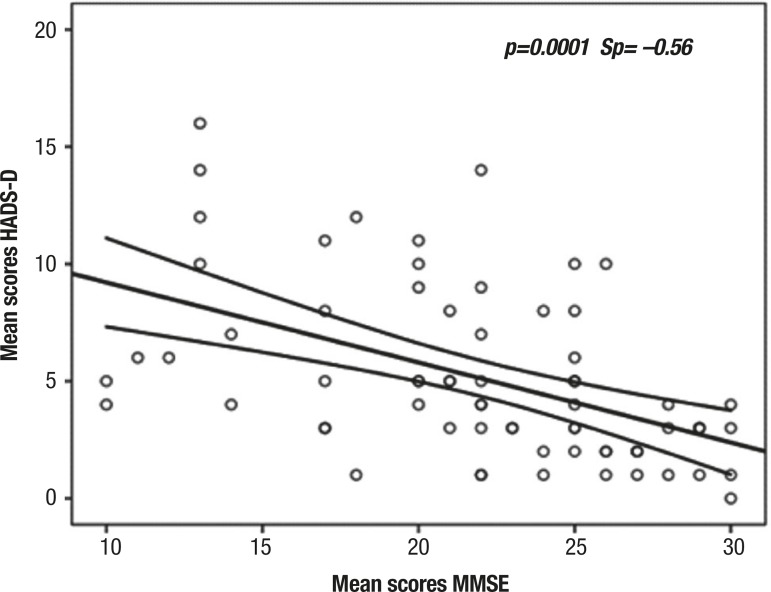
Correlation between mean scores on subscale of depressive symptoms (HADS-D) and on Mini-Mental State Examination (MMSE) in elderly patients hospitalized at the University Hospital Lauro Wanderley (n=70).

A moderate linear negative correlation was also found (p<0.05, Sp= -0.56) between mean scores on the HADS-D and MMSE, suggesting that the higher the scores on the HADS-D, the lower the scores on the MMSE (Figure 3). No correlation was observed between scores on the MMSE and on the HADS-A.

## DISCUSSION

The results observed in this study suggested a prevalence of delirium in elderly patients hospitalized within an educational public institution that was consistent with the literature.

Regarding age distribution, age groups in which we observed the highest frequencies are in line with evidence that, in general, there is a predominance of hospitalizations in younger elderly and a decline with increasing age.^24^ Low educational level of the patients in our sample is consistent with studies that assessed all-age inpatients from Brazilian public hospitals.^25,26^ Since educational level is associated with level of reasoning and that 67.1% of respondents were illiterate or had incomplete primary school, performance on the MMSE can be expected to be unreliable. To avoid biases related to education, different cutoffs were adopted according to the educational level of patients.

The prevalence of delirium in elderly patients varies widely across studies, depending on patient circumstances. While the rate ranges from 15% to 20% in elderly from clinical and surgical wards,^4,5^ it can be over 50% in individuals with hip fractures, and up to 80% among mechanically ventilated patients in the ICU.^3^ Differences in prevalence have also been explored by other studies. In a study of 104 hospitalized elderly medical patients in a teaching hospital,^27^ the prevalence of delirium was 18%, as assessed by CAM. The prevalence of delirium was expected to be higher in older patients, as observed in another prevalence study that found a higher rate in patients over 80 years old.

In a previous study, the prevalence of cognitive impairment among 224 elderly patients with a mean age of 71.5 years and mostly under-educated (74.6% with up to 4 years of education) was found to be 37.4%, much higher than the rate found in the present study.^28^ Another study conducted at the HULW, found a correlation between delirium and duration of hospital stay (25.6 days±16.7 days) in patients of all ages.^8^ We found no significant difference in hospital stay between patients with or without delirium.

Our sample, however, had few patients over 80 years old, possibly because the hospitalization rate is lower in patients older than 80 years in Brazil.^24^ Some authors suggest that the older old may be more resistant to depression, although no significant differences were observed among the youngest group (60-69 years), intermediate-age (70-79 years) and the oldest group (80 years or more).^29^

Although gender is thought to be an important variable in the prevalence of depression and depressive symptoms, there was no significant association in the present study. However, two previous Brazilian studies showed a higher prevalence among women.^30,31^ This gender influence is still controversial, where proposed explanations include sociocultural factors related to negative psychological experiences and greater susceptibility to stressful events.

However, the HADS-D and HADS-A are not independent: the average correlation in our study was 0.50 (Figure 2), unsurprising given the comorbid symptoms of anxiety and depression. Prevalence of depressive symptoms in elderly hospitalized patients evaluated in our study by the HADS-D was consistent with results from other international and Brazilian inpatient prevalence studies.

The depressive dimension is strongly marked by the measure of anhedonia (five of seven items), a characteristic of depression, and anxiety whose magnitude is measured by feelings of stress, worry, fear and panic (five of seven items). Depression itself is also often associated with anxiety, with which it overlaps symptomatically and is co-involved.^31,32^ This scale produces a composite score, but allows the differential analysis of the main groups of symptoms associated with depression. Other authors found high levels of depressive symptoms (56.7%) in 30 elderly patients admitted to a public hospital in Maringa, Parana.^29^

Depressive symptoms and delirium overlapped in 5.7% of our sample, similar to findings reported by other authors in 459 elderly patients hospitalized within a general medical service of a hospital, where 5.0% had the overlapping syndrome, 39 (8.5%) delirium alone, and 121 (26.3%) had depression without delirium.^33^ However, in another study of 277 elderly patients hospitalized with hip fracture (age 78.0±8.2 years), 10.8% had depressive symptoms alone, 31.8% delirium, 21.7% overlapping syndrome, and 35.7% had neither of the two conditions.^32^ The assessment of cognition is important to differentiate between delirium, dementia and depression. For these three syndromes, a large overlap may exist simultaneously in the same patient, and often confers increased risk for other disorders.^34^ Despite its clinical importance, delirium often goes unnoticed or is diagnosed as dementia or depression.^5^ Our data showed a significant association between cognitive status (assessed by MMSE) and the presence of delirium (p=0.001) in hospitalized elderly.

The prevalence of psychiatric disorders assessed in the present study does not necessarily indicate the existence of depression, and patients who have reached the scale cutoff should be examined using clinical diagnostic methods. The HADS is not a substitute for a diagnostic interview by mental health professionals, but a useful screening tool in the clinical setting to facilitate the assessment of depression in the elderly, especially when reference measurements are compared with subsequent scores.

Additionally, these results cannot be freely extrapolated to elders that are institutionalized or treated at specialized geriatric services, because our sample was small and involved patients from clinical and surgical wards of a university hospital.

It was concluded that there was significant occurrence of delirium during the late afternoon in these patients, possibly compatible with the sunset syndrome and associated with depressive symptoms and cognitive impairment, and without apparent relationship with infectious processes, fever, number of medications, length of hospital stay or anxiety symptoms. The use of protocols evaluating mental state, orientation and anxiety-depression symptoms in elderly patients can help identify patients at high risk of delirium in tertiary centers.
